# The Impact of a Preoperative Staging System on Accurate Prediction of Prognosis in Intrahepatic Cholangiocarcinoma

**DOI:** 10.3390/cancers14051107

**Published:** 2022-02-22

**Authors:** Hisashi Kosaka, Masaki Ueno, Koji Komeda, Daisuke Hokuto, Hiroya Iida, Fumitoshi Hirokawa, Kosuke Matsui, Mitsugu Sekimoto, Masaki Kaibori

**Affiliations:** 1 Department of Surgery, Kansai Medical University, Hirakata 573-1010, Japan; matsuik@hirakata.kmu.ac.jp (K.M.); sekimotm@hirakata.kmu.ac.jp (M.S.); kaibori@hirakata.kmu.ac.jp (M.K.); 2 Second Department of Surgery, Wakayama Medical University, Wakayama 641-8509, Japan; ma@wakayama-med.ac.jp; 3 Department of General and Gastroenterological Surgery, Osaka Medical and Pharmaceutical University, Takatsuki 569-8686, Japan; komeda0502@gmail.com (K.K.); fumitoshi.hirokawa@ompu.ac.jp (F.H.); 4 Department of Surgery, Nara Medical University, Kashihara 634-8521, Japan; hokuto@naramed-u.ac.jp; 5 Department of Surgery, Shiga University of Medical Science, Otsu 520-2192, Japan; hiroya@belle.shiga-med.ac.jp

**Keywords:** intrahepatic cholangiocarcinoma, cancer staging, tumor staging

## Abstract

**Simple Summary:**

Non-invasive biomarkers detected preoperatively are still inadequate for treatment decision making for patients with intrahepatic cholangiocarcinoma (ICC). In this study, we analyzed preoperative findings to establish a novel preoperative staging system (PRE-Stage) for patients with ICC. A newly invented PRE-Stage was developed using a CRP–albumin–lymphocyte index < 3, central tumor location, and CA19-9 level > 40 U/mL, and it was able to significantly predict DSS and DFS when the patients were stratified into four stages (*p* < 0.05). The PRE-Stage demonstrated similar accuracy in predicting the prognosis of ICC as that of the Liver Cancer Study Group of Japan stage, which is based on postoperative findings. The PRE-Stage may contribute to appropriate treatment decision making.

**Abstract:**

Background: Non-invasive biomarkers detected preoperatively are still inadequate for treatment decision making for patients with intrahepatic cholangiocarcinoma (ICC). In this study, we analyzed preoperative findings to establish a novel preoperative staging system (PRE-Stage) for patients with ICC. Methods: The clinical data of 227 consecutive patients with histologically confirmed ICC following hepatectomy at five university hospitals were analyzed. Results: Cox proportional hazards regression analysis of survival revealed that a CRP–albumin–lymphocyte index < 3, central tumor location, and CA19-9 level > 40 U/mL were prognostic factors among the preoperatively obtained clinical findings (hazard ratios (HRs) of all three factors for disease-specific survival (DSS) and disease-free survival (DFS: 2.4–3.3 and 1.7–2.9; all *p* < 0.05). The PRE-Stage was developed using these three prognostic factors, and it was able to significantly predict DSS and DFS when the patients were stratified into four stages (*p* < 0.05). In addition, the PRE-Stage resulted in similar HRs as those of the Liver Cancer Study Group of Japan (LCSGJ) stage (HRs for DSS: PRE-Stage, 1.985; LCSGJ stage, 1.923; HRs for DFS: LCSGJ stage, 1.909, and PRE-Stage, 1.623, all *p* < 0.05). Conclusion: The PRE-Stage demonstrated similar accuracy in predicting the prognosis of ICC as that of the LCSGJ stage, which is based on postoperative findings. The PRE-Stage may contribute to appropriate treatment decision making.

## 1. Introduction

Intrahepatic cholangiocarcinoma (ICC) is still a challenging neoplasm to treat, and its incidence has increased over the past three decades [1,2]. Surgery is considered a potentially curative treatment; however, the prognosis of advanced ICC is still dismal [3]. Preoperative estimation of prognosis may lead to appropriate indications for surgery and chemotherapy, whereas the existing cancer staging systems such as the Liver Cancer Study Group of Japan (LCSGJ) stage are based on histopathological findings obtained postoperatively [4,5,6]. Preoperative findings are useful non-invasive biomarkers. Many indices estimating nutrition, immunity, and inflammatory statuses, such as the neutrophil-to-lymphocyte ratio (NLR), prognostic nutritional index (PNI), platelet-to-lymphocyte ratio (PLR), C-reactive protein (CRP)-to-albumin ratio (CAR), and CRP–albumin–lymphocyte (CALLY) index, have been reported as acceptable prognostic factors for various types of carcinomas [7,8,9,10,11,12]. The preoperative intrahepatic tumor location can also predict prognosis in patients with ICC [13,14]. However, these non-invasive biomarkers, which are based on preoperative findings, are still inadequate to use for treatment decision making and life planning of the patients. In this study, we analyzed the preoperative findings of patients with ICC to detect accurate prognostic factors and, using these factors, we established a novel preoperative staging system (PRE-Stage) for practical use in patients with ICC.

## 2. Materials and Methods

### 2.1. Patients

We retrospectively analyzed the clinical and histopathologic data of 227 consecutive patients with histologically confirmed ICC following hepatectomy at five university hospitals in the Kansai region of Japan between January 2009 and December 2020. ICC was defined as tumor arises from intrahepatic bile duct in accordance with histological evaluation, whereas perihilar cholangiocarcinoma was excluded from this study cohort. Clinical data were collected from each hospital and then compiled and analyzed at Kansai Medical University. LCSGJ firstly proposed an independent ICC staging system in 1997. Subsequently, the 7th edition of AJCC staging was proposed to address ICC separately from HCC in 2010. In this study, most recent version of LCSGJ staging (6th edition) and American Joint Committee on Cancer (AJCC) staging (8th edition) were used for evaluating postoperative cancer stage of ICC [5,6].

### 2.2. Calculation of Indices Estimating Nutrition, Immunity, and Inflammatory Statuses

NLR was calculated as the absolute neutrophil count/absolute lymphocyte count (ALC) [8]. The PNI was calculated as 10 × albumin level + 0.005 × ALC [9]. The PLR was calculated as the platelet count/ALC [10]. The CAR was calculated as the CRP level/albumin level [11]. The CALLY index was calculated as (albumin level × ALC)/(CRP level × 10^4^) [12].

### 2.3. Intrahepatic Tumor Location-Specific Preoperative Categorization of ICC

Previously reported criteria for tumor location-dependent stratification of ICC was used as follows [13]. Computed tomography (CT) was performed to determine intrahepatic tumor location. The portal vein (PV) is a distinct landmark on CT images of the liver. The liver was divided into three areas based on the distance from the PV branches. The area within 10 mm of the first portion of the PV (right and left PV) was defined as the central area. The area within 10 mm from the second portion of the PV (i.e., the umbilical portion, anterior PV, and posterior PV), excluding the central area, was defined as the intermediate area. The area outside of the intermediate areas was defined as the peripheral area. ICC were classified as peripheral ICC, intermediate ICC, or central ICC, depending on the location of the innermost portion of the tumor relative to the hilus hepatis. At the time intrahepatic bile duct dilatation was observed without mass forming, such as a periductal infiltrating tumor, MRCP and/or ERCP were performed to identify the innermost portion of the tumor relative to the hilus hepatis.

### 2.4. Statistical Analysis

Data are expressed as numbers with percentages or medians with interquartile ranges. The Shapiro–Wilk test was used to assess the normality of continuous variables. Student’s t test or Welch’s test following Levene’s test was used for comparisons of normally distributed data, and the Mann–Whitney U test was used for comparisons of non-normally distributed data. Fisher’s exact test was used for comparisons of nominal variables. Comparisons were considered statistically significant at *p* < 0.05. The area under the receiver operating characteristic (ROC) curve (AUC) was calculated to compare the prognostic abilities of the variables. Youden’s index was used to identify cutoff values. The Kaplan–Meier method and log-rank test were performed to assess differences in disease-specific survival (DSS) and disease-free survival (DFS). DSS begins at the time of surgery and ends at the time of disease specific death. A total of 14 patients who died from other obvious reasons such as heart attack or cerebral hemorrhage were excluded from disease specific death. DFS was analyzed in 223 patients with R0 or R1 resection margins among the of 227 study patients; the 4 patients who had R2 resection margins were excluded from the analysis. Cox proportional hazards regression analysis was performed using a forward stepwise method to detect independent risk factors for DSS and DFS; in this analysis, continuous variables were transformed into binary variables based on the cutoff values detected by the ROC analysis. Hazard ratios (HRs) with 95% confidence intervals (CIs) were estimated. All statistical analyses were performed using IBM SPSS ver. 22 software package for Windows (IBM Japan Ltd., Tokyo, Japan).

### 2.5. Ethics

This study was approved by the institutional review board of Kansai Medical University (approval number: 2019322) and was performed in accordance with the Declaration of Helsinki.

## 3. Results

### 3.1. Background Characteristics of the Study Cohort

The background characteristics of the 227 consecutive patients with histologically confirmed ICC following hepatectomy at five university hospitals in the Kansai region of Japan between January 2009 and December 2020 are summarized in Table 1. In this study cohort, the median age was 72 years, and the majority of patients were male (69.6%). Hepatobiliary data were within the normal ranges, and the median indocyanine green (ICG) was 10.0%. The median score of the albumin-bilirubin (ALBI) score was −2.82 and the median value of fibrosis-4 (FIB-4) index was 2.05. The median CA19-9 level was slightly higher than the reference limit (44.0 U/mL). The median values of the indices used to estimate nutrition status were as follows: NLR, 2.5; PNI, 48.2; PLR, 140.0; CAR, 0.04; and CALLY index, 3.7. The proportion of patients with a central tumor location was 34.8%.

### 3.2. Comparisons of the Prognostic Abilities of Indices Estimating Nutrition, Immunity, and Inflammatory Statuses

**Figure 1:**
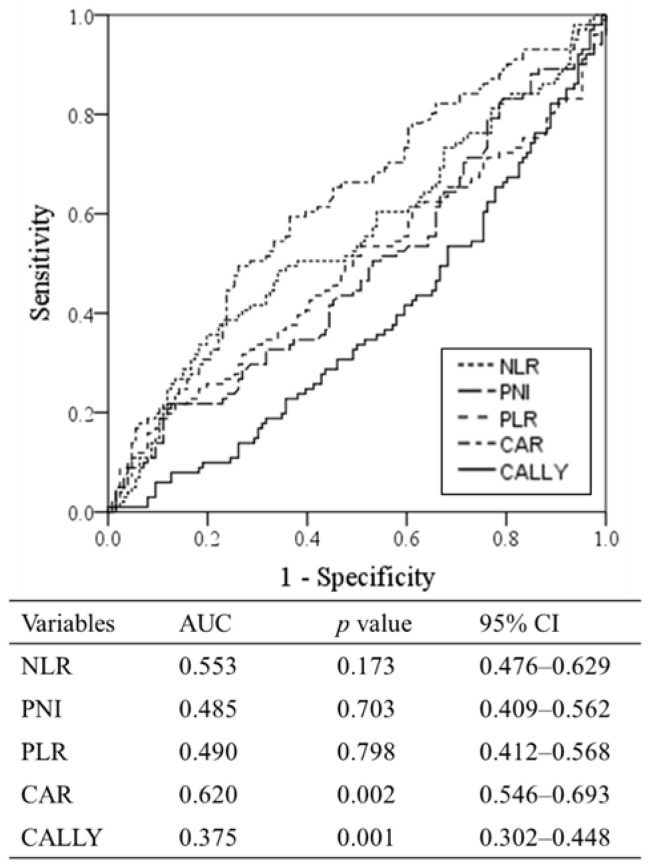
Comparisons of the prognostic ability of indices estimating nutrition, immunity, and inflammatory statuses. Area under the receiver operating characteristic curve (AUC) comparing the prognostic abilities of indices estimating nutrition, immunity, and inflammatory statuses. NLR: neutrophil-to-lymphocyte ratio, PNI: prognostic nutritional index, PLR: platelet to lymphocyte ratio, CAR: C-reactive protein-to-albumin ratio, CALLY index: CRP–albumin–lymphocyte index, AUC: area under the curve, and CI: confidence interval.

The ability to predict DSS of indices estimating nutrition, immunity, and inflammatory statuses (i.e., NLR, PNI, PLR, CAR, and CALLY index) were compared by ROC analysis (Figure 1). As a result, the CALLY index demonstrated the highest ability to predict DSS (AUC: 0.375, *p* = 0.001) at a cutoff value of 3.00, and the CAR demonstrated the second highest prognostic ability (AUC: 0.620, *p* = 0.002). However, the NLR, PNI, and PLR did not demonstrate significant prognostic value (*p* < 0.05).

### 3.3. Preoperative Prognostic Factors Associated with Disease-Specific Survival

Cox proportional hazards regression analysis of DSS and DFS was performed to detect prognostic factors among eight preoperatively obtained findings (Table 2). The CALLY index was chosen as one of the eight variables among indices estimating nutrition, immunity, and inflammatory statuses based on the results of ROC analyses (Figure 1). As a result, central tumor location, CALLY index < 3, and CA19-9 level > 40.05 U/mL demonstrated significantly higher HRs for DSS (3.292, 3.038, and 2.403, respectively, all *p* < 0.05). Age > 71.5 years and hepatitis and ICG < 9.65% also demonstrated statistical significance, but the HRs were lower compared with those for central tumor location, CALLY index, and CA19-9 level.

Regarding DFS, a CALLY index < 3, central tumor location, and CA19-9 level > 40.05 U/mL demonstrated higher HRs for DFS (2.937, 2.262 and 1.733, respectively, all *p* < 0.05) compared with age > 71.5 years and hepatitis and ICG < 9.65%. These results indicated that a CALLY index < 3, central tumor location, and CA19-9 level > 40 U/mL were strong prognostic factors among the preoperatively obtained findings in ICC patients.

### 3.4. Effects of Preoperatively Obtained Prognostic Factors on Patient Survival

**Figure 2:**
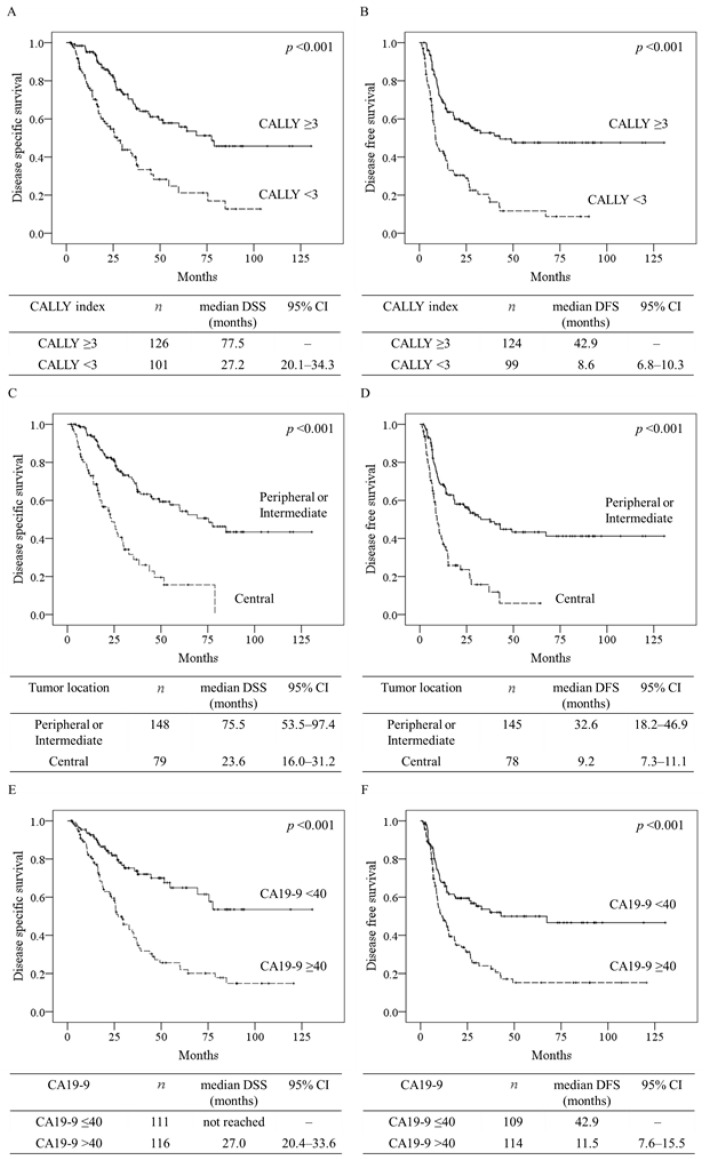
Ability of preoperatively obtained prognostic factors to predict patient survival. The results of Kaplan–Meier analysis and log-rank tests assessing disease-specific survival (**A**,**C**,**E**) and disease-free survival (**B**,**D**,**F**) according to the prognostic factors CALLY index < 3 (**A**,**B**), central tumor location (**C**,**D**), and CA19-9 level > 40 U/mL (**E**,**F**). CALLY index: CRP–albumin–lymphocyte index, CA19-9: carbohydrate antigen 19-9, DSS: disease-specific survival, DFS: disease-free survival, and CI: confidence interval.

The results of Kaplan–Meier analysis and log-rank tests assessing DSS and DFS according to three prognostic factors (CALLY index < 3, central tumor location, or CA19-9 level > 40 U/mL) are shown in Figure 2. CALLY index < 3, central tumor location, or CA19-9 level > 40 U/mL were all associated with a significantly poor DSS (median: 27.2, 23.6, and 27.0 months, respectively, all *p* < 0.05) and DFS (median: 8.6, 9.2, and 11.5 months, respectively, all *p* < 0.05).

### 3.5. Establishment and Impact of the PRE-Stage

**Figure 3:**
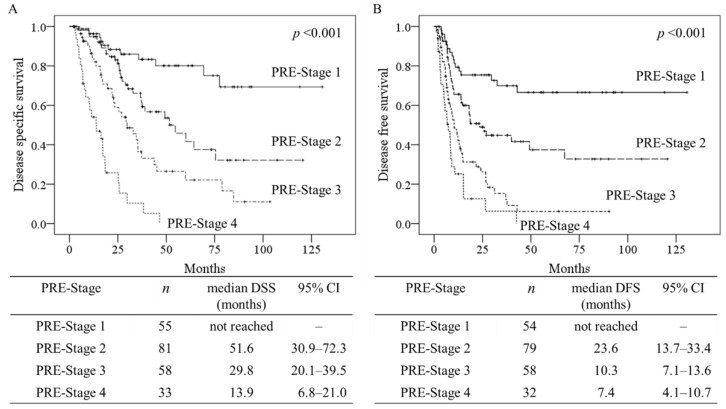
Impact of the preoperative staging system on patient survival. The results of Kaplan–Meier analysis and log-rank tests assessing disease-specific survival (**A**) and disease-free survival (**B**) according to the preoperative staging system. PRE-Stage, preoperative staging system, DSS: disease-specific survival, DFS: disease-free survival, and CI: confidence interval.

We hypothesized that the CALLY index < 3, central tumor location, and CA19-9 level > 40 U/mL are candidate prognostic factors for establishing a PRE-Stage for ICC. The PRE-Stage criteria are described in Table 3. The study cohort was divided into four stages according to the presence of these criteria, as shown in Figure 3. When Kaplan–Meier curves for DSS were stratified by the PRE-Stage, the differences among all stages were significant (all *p* < 0.05) (Figure 3A). The same was true for DFS (all *p* < 0.05) (Figure 3B). In addition, Cox proportional hazards regression analysis of DSS and DFS was performed to determine the HRs for each prognostic factor (Table 4). LCSGJ stage is one of representative staging system which based on histopathological findings obtained postoperatively. Both the PRE-Stage and LCSGJ stage demonstrated significantly high HRs for DSS (PRE-Stage vs. LCSGJ stage: 1.985 vs. 1.923, all *p* < 0.05) and DFS (1.909 vs. 1.623, all *p* < 0.05). The HRs were similar between the PRE-Stage and LCSGJ staging system.

### 3.6. Comparisons of Patient Characteristics Stratified by PRE-STAGE

Differences in the patient characteristics according to the PRE-Stage are shown in Table 5. Of the three ICC prognostic factors, the CA19-9 level (PRE-Stage 1 vs. 4: 12.0 vs. 712.0 U/mL, *p* < 0.05) and rate of central tumor location (PRE-Stage 1 vs. 4: 0% vs. 100.0%, *p* < 0.05) increased with the PRE-Stage, whereas the CALLY index decreased (PRE-Stage 1 vs. 4: 7.3 vs. 1.0, *p* < 0.05). In terms of tumor characteristics, the tumor size, rate of vascular invasion or main bile duct invasion, and rate of lymph node metastasis all increased significantly with advancing PRE-Stage (all *p* < 0.05). As a result, an advanced LCSGJ stage was frequently observed in patients with a more advanced PRE-Stage (*p* < 0.05)

## 4. Discussion

Many indices estimating nutrition, immunity, and inflammatory statuses, such as the NLR, PNI, PLR, CAR, and CALLY index, have been reported as acceptable prognostic factors for different types of carcinomas [7,8,9,10,11,12]. In this study, we compared the ability of these indices to predict DSS in patients with ICC by ROC analysis. As a result, the CALLY index demonstrated the highest prognostic ability in patients with ICC (AUC: 0.375, *p* = 0.001). In addition, there was a significant difference in the median DSS when the patients were stratified by the CALLY index cutoff (<3 vs. ≥3: 27.2 vs. 77.5 months, *p* < 0.05). Even though the CALLY index is a new index for estimating nutrition, immunity, and inflammatory statuses, very recently established in 2021 based on patients with hepatocellular carcinoma, it demonstrated a significant impact on prognosis in patients with ICC.

In this study, Cox proportional hazards regression analysis identified a CALLY index < 3, central tumor location, and CA19-9 level > 40 U/mL as prognostic factors among preoperatively obtained clinical findings in patients with ICC (HRs for DSS: central tumor location, 3.292; CALLY index, 3.038; CA19-9 level, 2.403; HRs for DFS: CALLY index, 2.937; central tumor location, 2.262; and CA19-9 level, 1.733, all *p* < 0.05). Even though the CAR also demonstrated a high ability to predict DSS according to ROC analysis, the CALLY index was used alone in the Cox proportional hazards regression analysis to avoid collinearity.

Using these three prognostic factors, the preoperative staging system (PRE-Stage) was constructed. PRE-Stage is uniquely based on non-invasive biomarkers and the radiologically diagnosed intrahepatic tumor location. Of these biomarkers, the CALLY index is determined by the serum albumin value as an indicator of nutrition status and liver function, the lymphocyte count as an indicator of immune status, and the CRP level as an indicator of inflammation level [12]. On the other hand, an intrahepatic central tumor location represents local tumor aggressiveness, such as large tumor size, frequent lymph node metastasis, and vascular and main bile duct invasion [13]. Another non-invasive biomarker, the CA19-9 level, reflects the systemic aggressiveness of tumor [15]. Taken together, the PRE-Stage reflects the nutrition, immunity, and inflammatory statuses of a patient and tumor aggressiveness simultaneously in patients with ICC.

This newly developed preoperative staging system demonstrated significant differences in survival depending on the PRE-Stage, according to the Kaplan–Meier method and log-rank test (survival according to stage, all *p* < 0.05). The rates of vascular invasion or main bile duct invasion and lymph node metastasis increased with advancing PRE-Stage (all *p* < 0.05). In addition, the PRE-Stage demonstrated similar prognostic ability to that of the LCSGJ stage, which is determined based on postoperative findings (HRs for DSS of PRE-Stage vs. LCSGJ stage: 1.923 vs. 1.985, all *p* < 0.05). Precise preoperative prediction of prognosis can contribute to better life planning and treatment decision making for ICC patients. When DSS was stratified by the PRE-Stage, the median DSS of patients with PRE-Stage 4 was 13.9 months. As the median survival time of patients with unresectable biliary tract cancer treated with gemcitabine plus cisplatin was 11.7 months in the ABC-02 trial [16], the surgical indications should be carefully determined for patients with PRE-Stage 4. In addition, when DFS was stratified by PRE-Stage, a majority of patients with PRE-Stage 3 or 4 developed early recurrence, within a year (median DFS of PRE-Stage 3 vs. 4 patients: 10.3 vs. 7.4 months). Such patients with poor prognoses may be candidates for neoadjuvant chemotherapy in future clinical trials, whereas few retrospective studies have evaluated neoadjuvant chemotherapy for patients with locally advanced ICC [17]. On the other hand, PRE-Stage 1 was associated with significantly better survival (median DSS: not reached) compared with the advanced stages. The histopathological characteristics of PRE-Stage 1 patients are small tumor size (median: 30 mm), mass forming type (83.6%), and lower rate of lymph node metastasis (15.8%). Recently, improvements in surgical techniques and perioperative management have extended the indications for hepatectomy in elderly patients [18,19]. Thus, hepatectomy may be considered in any patient diagnosed as PRE-Stage 1, even those who are elderly or high risk. In addition, such patients with better prognoses may be excluded from future trials of neoadjuvant chemotherapies.

## 5. Limitations

This study was limited by its retrospective and non-randomized nature. In spite of being a multicenter center study based over 12 years, the study cohort is still small and potentially biased. Even though HRs of three prognostic factors (CALLY index < 3, central tumor location, and CA19-9 level > 40 U/mL) in the Cox proportional hazards regression analysis were slightly different in the range of 2.4 to 3.2 for DSS, the factors were counted as same value of determinants of stage in the PRE-Stage system. External validation or prospective verification may be required to validate the accuracy of the proposed PRE-Stage. There is likely a subset of patients with elevated CA19-9 levels due to biliary obstruction, whereas jaundice is less frequent in patients with ICC. Another limitation of this study is that approximately 5–10% of individuals are Lewis antigen negative with scarce secretion of CA19-9. Patients without Lewis antigens have a potential to underestimate in PRE-Stage.

## 6. Conclusions

In this study, we developed a new preoperative staging system for patients with ICC. The PRE-Stage is uniquely based on non-invasive biomarkers and a radiologically diagnosed intrahepatic tumor location. Preoperative and accurate prediction of prognosis may contribute to better life planning and appropriate therapeutic strategies for patients with ICC.

## Figures and Tables

**Table 1:** Background characteristics.

Variable	*N* (%) or Median (IQR)
*N*	227
Age	72.0 (66.0–77.0)
Sex, male	158 (69.6)
HBV/HCV	35 (15.4)/22 (9.7)
Neutrophil count	3550.7 (2692.5–4633.5)
Lymphocyte count	1417.3 (1140.7–1865.1)
Albumin, g/dL	4.1 (3.8–4.4)
Total bilirubin, mg/dL	0.7 (0.5–0.9)
AST, U/L	27.0 (22.0–35.0)
ALT, U/L	23.0 (16.0–31.8)
ALP, U/L	301.5 (230.5–455.0)
CRP, mg/dL	0.16 (0.08–0.45)
Platelet count, ×10^4^ μL	20.7 (15.7–25.7)
ALBI score	−2.82 (−3.04–−2.48)
FIB4 index	2.05 (1.48–2.85)
ICG, %	10.0 (7.0–13.9)
CEA, ng/mL	2.9 (1.9–4.9)
CA19-9, U/mL	44.0 (14.3–256.7)
NLR	2.5 (1.8–3.2)
PNI	48.2 (44.3–52.0)
PLR	140.0 (99.0–179.2)
CAR	0.04 (0.02–0.12)
CALLY index	3.7 (1.3–8.7)
Tumor location: Peripheral/Intermediate/Central	63/85/79 (27.8/37.4/34.8)

HBV Hepatitis B virus, HCV Hepatitis C virus, AST Aspartate transaminase, ALT Alanine transaminase, ALP Alkaline phosphatase, CRP C-reactive protein, ALBI score Albumin–bilirubin score, FIB4 index Fibrosis-4 index, ICG Indocyanine green, CEA Carcinoembryonic antigen, CA19-9 Carbohydrate antigen 19-9, NLR Neutrophil to lymphocyte ratio, PNI Prognostic nutritional index, PLR Platelet to lymphocyte ratio, CAR C-reactive protein to albumin ratio, and CALLY index CRP–albumin–lymphocyte index.

**Table 2:** COX proportional hazards regression analysis of the prognostic ability of the preoperative findings.

Variable	Disease-Specific Survival		Disease-Free Survival	
	Hazard Ratio (95% CI)	*p* Value	Hazard Ratio (95% CI)	*p* Value
Age > 71.5 years	1.751 (1.102–2.782)	0.018	1.653 (1.121–2.438)	0.011
Sex, Male	–	0.857	–	0.733
Hepatitis, positive	1.693 (1.260–2.275)	<0.001	1.533 (1.163–2.019)	0.002
ICG < 9.65%	1.570 (1.028–2.399)	0.037	1.535 (1.059–2.224)	0.024
CALLY index < 3.00	3.038 (1.951–4.730)	<0.001	2.937 (1.991–4.334)	<0.001
CA19-9 > 40.05 U/mL	2.403 (1.525–3.788)	<0.001	1.733 (1.171–2.564)	0.006
CEA > 4.35 ng/mL	–	0.639	–	0.803
Tumor location, central	3.292 (2.061–5.260)	<0.001	2.262 (1.505–3.400)	<0.001

CI: Confidence interval, ICG: Indocyanine green, CALLY index: CRP–albumin–lymphocyte index, CA19-9: Carbohydrate antigen 19-9, and CEA: Carcinoembryonic antigen.

**Table 3:** Criteria of the preoperative staging system (PRE-Stage).

Determinants of stage CALLY index < 3 CA19-9 level > 40 U/mL Tumor location, central
PRE-Stage 1: ALL negative PRE-Stage 2: One determinant is positive PRE-Stage 3: Two determinants are positive PRE-Stage 4: All three determinants are positive
CALLY index CRP–albumin–lymphocyte index, CA19-9 Carbohydrate antigen 19-9, PRE-Stage Preoperative staging system

**Table 4:** COX proportional regression hazards analysis of prognostic ability.

Variable	Disease-Specific Survival		Disease-Free Survival	
	Hazard Ratio (95% CI)	*p* Value	Hazard Ratio (95% CI)	*p* Value
CALLY index < 3.00	–	0.841	–	0.309
CA19-9 > 40.05 U/mL	–	0.978	–	0.518
Tumor location, central	–	0.813	–	0.673
PRE-Stage	1.923 (1.521–2.433)	<0.001	1.623 (1.329–1.982)	<0.001
LCSGJ stage	1.985 (1.564–2.519)	<0.001	1.909 (1.535–2.375)	<0.001
AJCC stage	–	0.348	–	0.647

CI Confidence interval, ICG Indocyanine green, CALLY Index CRP–albumin–lymphocyte index, CA19-9 Carbohydrate antigen 19-9, PRE-Stage Preoperative staging system, LCSGJ Liver Cancer Study Group of Japan, AJCC American Joint Committee on Cancer.

**Table 5:** Patient characteristics according to the preoperative staging system (PRE-Stage).

Variable		PRE-Stage				
	All	PRE-Stage 1	PRE-Stage 2	PRE-Stage 3	PRE-Stage 4	*p* Value
N	227	55	81	58	33	–
Age	72.0 (66.0–77.0)	73.0 (69.0–75.5)	71.0 (65.0–77.0)	71.0 (67.8–78.3)	71.0 (64.0–74.0)	0.555
Sex, Male	158 (69.6)	42 (76.4)	58 (71.6)	35 (60.3)	23 (69.7)	0.297
ICG, %	10.2 (7.0–14.0)	10.0 (7.0–14.0)	10.0 (7.0–13.4)	10.0 (7.0–14.5)	10.8 (8.1–14.7)	0.734
CEA, ng/mL	2.9 (1.9–4.9)	2.4 (1.6–3.7)	3.0 (1.8–4.6)	2.8 (2.3–5.4)	6.0 (2.8–24.1)	<0.001
CA19-9, U/mL	44.0 (145–234)	12.0 (6–23)	42.0 (13–122)	98.9 (30–689)	712.0 (179–5053)	<0.001
CALLY index	3.7 (1.3–8.6)	7.3 (4.5–17.0)	5.0 (2.2–11.8)	1.8 (0.5–20.5)	1.0 (0.2–2.2)	<0.001
Tumor location, central	79 (34.8)	0 (0.0)	13 (16.0)	33 (56.9)	33 (100.0)	<0.001
Tumor size (mm)	35.0 (25.0–57.5)	30.0 (20.0–40.5)	35.0 (25.0–50.0)	39.0 (26.8–74.3)	60.0 (35.0–85.0)	<0.001
Multiple tumors	34 (15.0)	5 (9.1)	9 (11.1)	11 (19.0)	9 (27.3)	0.069
Tumor type, MF/PI/IG	178/35/14 (78.4/15.4/6.2)	46/6/3 (83.6/10.9/5.5)	67/7/7 (82.7/8.6/8.6)	44/12/2 (75.9/20.7/3.4)	21/10/2 (63.6/30.3/6.1)	0.066
Differentiation, Well/Mod/Por/Other	64/124/24/15 (28/55/11/7)	19/24/7/5 (35/56/12/9)	19/45/10/7 (24/56/12/9)	20/29/6/3 (35/50/10/5)	6/26/1/0 (18/79/3/0)	<0.001
Vascular invasion or main bile duct invasion	156 (68.7)	22 (40.0)	53 (65.4)	49 (84.5)	32 (97.0)	<0.001
Lymph node metastasis	43/114 (37.7)	3/19 (15.8)	3/27 (11.1)	21/39 (53.8)	16/29 (55.2)	<0.001
Resection margin, positive	31 (13.7)	3 (5.5)	9 (11.1)	10 (17.2)	9 (27.3)	0.024
6th LCSGJ stage, I/II/III/IVa/IVb	18/65/81/51/12 (8/29/36/23/5)	8/23/17/7/0 (15/42/31/13/0)	7/28/34/10/2 (9/35/42/12/3)	3/10/20/21/4 (5/17/35/36/7)	0/4/10/13/6 (0/12/30/39/18)	<0.001

PRE-Stage: Preoperative staging system, ICG: Indocyanine green, CEA: Carcinoembryonic antigen, CA19-9: Carbohydrate antigen 19-9, CALLY Index: CRP–albumin–lymphocyte index, MF: Mass forming type, PI: Periductal infiltrating type, IG: Intraductal growth type, Well: Well differentiated, Mod: Moderately differentiated, Por: Poorly differentiated, and LCSGJ: Liver Cancer Study Group of Japan.

## Data Availability

The data presented in this study are available on request from the corresponding author.
